# Anti-Ma2-antibody-associated encephalitis: An atypical paraneoplastic neurologic syndrome

**DOI:** 10.4102/sajr.v22i1.1310

**Published:** 2018-05-17

**Authors:** Bogna Targonska, Jamie Frost, Sanjay Prabhu

**Affiliations:** 1 Department of Radiology, Renown Regional Medical Center, Reno, NV, United States; 2 Helen DeVos Childrens Hospital, Advanced Radiology Services, Grand Rapids, MI, United States; 3 Department of Radiology, Boston Children’s Hospital, Boston, MA, United States

## Abstract

Paraneoplastic syndromes are a heterogeneous group of conditions affecting cancer patients, where the signs and symptoms are not owing to the local effects of the tumour but instead owing to humoral or immunologic effects. We describe an unusual presentation of a paraneoplastic neurologic syndrome presenting with predominant involvement of the hypothalamus and deep grey nuclei secondary to an anterior mediastinal germinoma and associated with anti-Ma2 antibody.

## Introduction

Paraneoplastic syndromes may affect multiple organ systems and may cause metabolic, endocrine, dermatologic, haematologic and neurologic derangements. Paraneoplastic neurologic syndrome (PNS) is a rare entity and can affect various areas of the central or peripheral nervous system. Onset of symptoms may in some instances precede the diagnosis of the underlying malignancy. Furthermore, findings on imaging of the brain may be the first step in the patient’s workup and consideration of this entity is important in further management.

## Case

An adolescent male presented to the emergency department with prolonged fatigue progressing over 5 months. This was followed by subjective fevers, decreased memory and increased confusion in the few days prior to presentation and finally an episode of hallucinations the evening prior to presentation.

The patient had no prior medical or surgical history and no known allergies. Vaccinations were up to date. He denied smoking, drinking or illicit drug use.

On examination in the emergency department, the patient was somnolent, but rousable, and answered questions appropriately. There was no focal neurological deficit.

Cell count and chemistry panel were unremarkable. Lumbar puncture revealed normal opening pressures, lymphocytic pleocytosis and an elevated protein level. Cerebrospinal fluid was negative for Ebstein–Barr Virus (EBV), cytomegalovirus (CMV), herpes simplex virus (HSV), tumour markers and anti-ACE (sarcoid). Oliogoclonal bands were positive, and an expanded work up for autoimmune causes and paraneoplastic processes was initiated.

**FIGURE 1:**
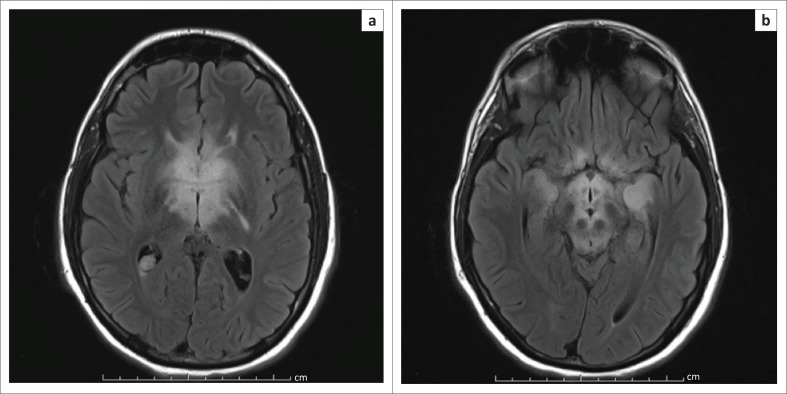
(a) Axial Fluid Attenuation Inversion Recovery (FLAIR) imaging demonstrating symmetric increased signal involving the basal ganglia and thalami bilaterally, (b) Increased signal intensity is also noted in the hypothalamus, anterior midbrain and to varying degrees in both medial temporal lobes.

**FIGURE 2:**
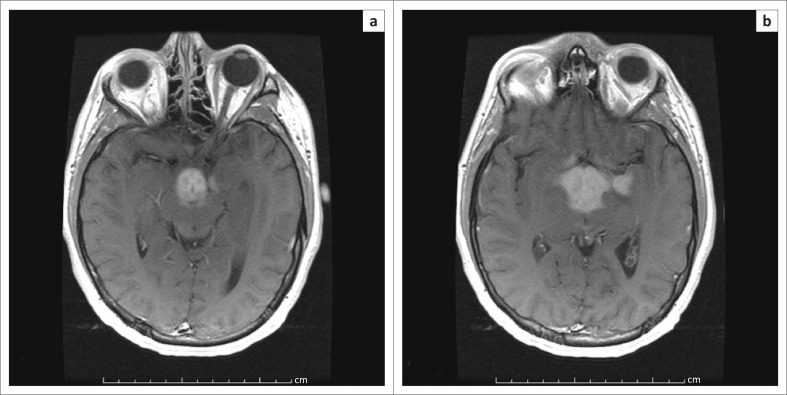
Axial T1-weighted post-contrast imaging (a and b) demonstrating avid enhancement corresponding to the regions of T2/FLAIR signal abnormality.

Magnetic resonance imaging (MRI) demonstrated abnormal T2 signal in the basal ganglia, thalami, hypothalamus and anterior midbrain. Increased signal was also noted involving both medial temporal lobes, left greater than right (Figure 1). These regions showed avid enhancement (Figure 2).

**FIGURE 3:**
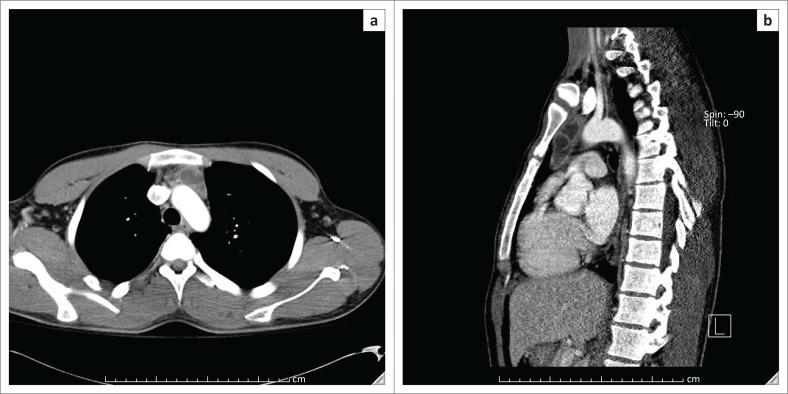
Axial (a) and sagittal (b) contrast enhanced computed tomography imaging of the chest demonstrates a well-defined cystic and solid mass containing punctate calcifications in the anterior mediastinum, without mass effect on the adjacent vascular structures.

Given the high clinical suspicion for a paraneoplastic process, further imaging was obtained including a scrotal ultrasound and computed tomography (CT) chest. The ultrasound was negative, but the CT demonstrated an anterior mediastinal mass (Figure 3).

The patient underwent resection of the anterior mediastinal mass. Analysis of the pathology specimen revealed a 4-cm germinoma associated with a thymic epithelial cyst. The paraneoplastic panel results became available at that time and demonstrated positive Anti-Ma2 antibodies.

**FIGURE 4:**
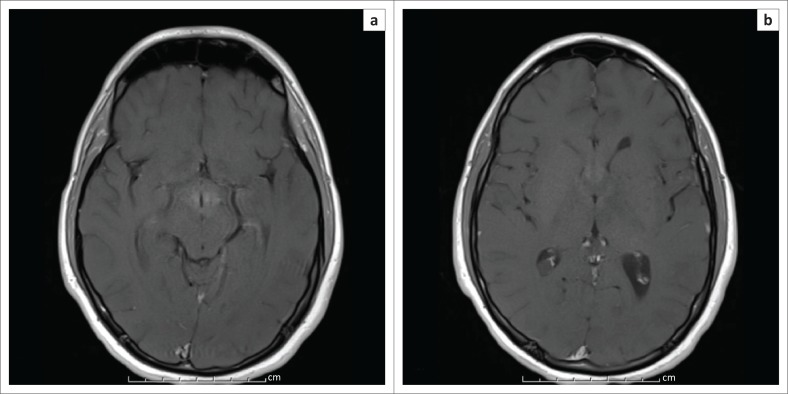
T1-weighted post-contrast imaging (a and b) following surgery demonstrates near-complete resolution of the previously noted enhancement.

Follow-up imaging on day 7 after surgery demonstrated persistence of T2/FLAIR abnormality, but a significant reduction in the degree of enhancement compared to pre-operative imaging (Figure 4).

## Discussion

Paraneoplastic neurological syndromes occur in patients with cancer and cause a wide range of clinical symptoms. Damage to the nervous system is thought to result from immunologic cross-reactivity between neoplastic antigens and normal neuronal tissues. The most common PNS is the Lambert-Eaton myasthenic syndrome (LEMS), which occurs in 2% – 3% of patients with small-cell lung cancer.^1^ Overall, PNS affect less than 1% of cancer patients and are rare compared to the direct effects of the tumour, its metastases or the effects of cancer treatment.^1,2^ Therefore, a diagnosis of a paraneoplastic syndrome should be made after the exclusion of other causes for the patient’s symptoms.

Fifty per cent of primary tumours associated with paraneoplastic limbic encephalitis are small-cell lung carcinomas.^3^ Other tumours associated with PNS include testicular germ cell tumours, breast cancer, thymoma, lymphoma and teratoma.^4^

Anti-Ma2-associated encephalitis is a PNS characterised by isolated or combined limbic, diencephalic or brainstem dysfunction.^5,6^ These antibodies can be detected in serum and cerebrospinal fluid and are highly specific for this disease entity.

Clinicopathologic features of paraneoplastic limbic encephalitis are well-defined, with patients usually presenting with short-term memory loss, seizures, irritability, depression and cognitive decline.

However, less than 30% of patients with anti-Ma2 encephalitis exhibit the typical clinical presentation of limbic encephalitis, and therefore this entity may go unrecognised for a prolonged period of time before the diagnosis is made.^1,2^ Patients may present with a wide array of symptoms, including excessive sleepiness, ocular movement abnormalities, hypokinesis or pure psychiatric disturbance.^5,6^ A series published by Dalmau et al. included 38 patients with anti-Ma2-associated encephalitis, where neurological symptoms preceded tumour diagnosis in 62% of patients.

Areas of the brain involved on MRI may include a combination of the medial temporal lobes, hypothalamus, basal ganglia, thalami or the upper brainstem. The regions of abnormality demonstrate increased signal on T2WI/FLAIR sequences and may occasionally enhance.

Early identification and treatment of the underlying malignancy is the most important component of treatment of the paraneoplastic syndrome. Unlike many of the other paraneoplastic syndromes, anti-Ma2-associated encephalitis has a relatively high response rate to treatment, with approximately 30% demonstrating neurological improvement and another 20% – 40% stabilising in response to therapy.^5,6^ In our case, the diagnosis of paraneoplastic encephalitis was made based on imaging findings, presence of anti-Ma2 antibodies and exclusion of other causes.

## Conclusion

We have presented an unusual case of a paraneoplastic neurological syndrome presenting with primarily hypothalamic and basal nuclei involvement and associated with anti-Ma2 antibodies where the tumour was found secondarily. This emphasises the need to consider rare diagnoses such as PNS when faced with unusual neurologic findings.
